# Predicting shockwave lithotripsy outcome for urolithiasis using clinical and stone computed tomography texture analysis variables

**DOI:** 10.1038/s41598-019-51026-x

**Published:** 2019-10-11

**Authors:** Helen W. Cui, Mafalda D. Silva, Andrew W. Mills, Bernard V. North, Benjamin W. Turney

**Affiliations:** 1 Oxford Stone Group, Nuffield Department of Surgical Sciences, University of Oxford, Oxford, United Kingdom; 2 University of Lisbon, Faculty of Sciences, Lisbon, Portugal; 3 Exploristics Ltd., Belfast, United Kingdom

**Keywords:** Urogenital diseases, Risk factors, Urology

## Abstract

We aimed to develop and evaluate a statistical model, which included known pre-treatment factors and new computed tomography texture analysis (CTTA) variables, for its ability to predict the likelihood of a successful outcome after extracorporeal shockwave lithotripsy (SWL) treatment for renal and ureteric stones. Up to half of patients undergoing SWL may fail treatment. Better prediction of which cases will likely succeed SWL will help patients to make an informed decision on the most effective treatment modality for their stone. 19 pre-treatment factors for SWL success, including 6 CTTA variables, were collected from 459 SWL cases at a single centre. Univariate and multivariable analyses were performed by independent statisticians to predict the probability of a stone free (both with and without residual fragments) outcome after SWL. A multivariable model had an overall accuracy of 66% on Receiver Operator Curve (ROC) analysis to predict for successful SWL outcome. The variables most frequently chosen for the model were those which represented stone size. Although previous studies have suggested SWL can be reliably predicted using pre-treatment factors and that analysis of CT stone images may improve outcome prediction, the results from this study have not produced a useful model for SWL outcome prediction.

## Introduction

Shockwave lithotripsy (SWL) has been proven to be an effective treatment for renal tract calculi^1,2^. Published SWL success rates range from 35% to 89%^1,3,4^. This variation is likely attributed to differences in clinical practice, and to inconsistent clinical outcome measures of success across the field. For stones less than 10 mm in size in the ureter, or non-lower pole positions of the kidney, SWL or ureteroscopy are the preferred treatment options. For renal stones between 10 mm and 20 mm, SWL or percutaneous nephrolithotomy or ureteroscopy is recommended except for lower pole renal stones where SWL is second line if there are unfavourable factors for SWL^5,6^. It was previously thought that ureteroscopy, as the alternative to SWL, had superior stone clearance rates to SWL with published success rates of between 85–95%^7^. However, recent prospective studies, which have used CT imaging to measure the rate of being completely stone free after ureteroscopy, have found a much lower success rate of 38–54%^8^. A recent evidence review conducted as part of the National Institute of Health and Care Excellence (NICE) guidelines on renal and ureteric stones concluded that there was only small benefit of ureteroscopy for stone removal over SWL for ureteric stones less than 10 mm; and that the clinical and cost effectiveness between SWL and ureteroscopy for renal stones less than 10 mm favoured SWL as the first line treatment choice^9^. Therefore, we need to improve the efficacy of these stone treatments. The ability to identify which stone cases will be unlikely to be successfully treated with SWL, is one method of increasing the efficacy of SWL.

Several studies have investigated factors that may be used in a predictive model for successful SWL^4,10–25^. Patient factors associated with decreased probability of SWL success include increasing patient age^4,10,13,16^, body mass index (BMI) or skin-to-stone distance (SSD)^11–13,21^, longer infundibular length^12^, ureteropelvic junction diameter^13^, and female gender^11,13^. Other unfavourable stone characteristics include higher Hounsfield unit (HU) density^11,15,18,21^, larger stone diameter and volume and greater number of stones^4,12,13,15,20,21,23^, greater stone heterogeneity^15,22^ and stone location in the kidney compared to the ureter^4,17^. Technical factors such as frequency of shock waves used, energy levels, accuracy of targeting the stone, focus size and patient breathing patterns will also affect SWL efficacy^5^.

Drawbacks of these previous studies include: the lack of consideration of all the potential factors together; the loss of the information from categorizing continuous variables; and the inaccurate and inconsistent measurement of stone parameters on imaging. To improve characterisation of stone heterogeneity as a predictor variable, we have used a software programme validated for measuring tumour heterogeneity^26^. This software performs CT textural analysis (CTTA) on every pixel within the area of the stone to give measurements of mean HU and total number of pixels, as well as statistical analysis of the nature of the distribution of the different HU values within the stone^27^.

We aimed to improve on the methodology of previous studies by including a comprehensive selection of both patient and imaging variables thought to influence SWL success; and by using multivariable analysis methods - other than logistic regression - to improve variable selection and reduce overfitting of the model.

## Results

### Patient characteristics

459 consecutive SWL cases (from 420 patients between 2011 and 2016 treated at a single centre) were included for analysis. Patient and stone characteristics are outlined in Table 1. BMI data was available in 176 cases (out of 459) and mean BMI was 28.1 ± 5.2 kg/m^2^. There was a weak correlation between BMI and SSD (Pearson r = 0.421). 41 cases (8.9%) had previous urological intervention including 22 cases of previous SWL in the same kidney, 9 cases of previous ureteroscopy or percutaneous nephrolithotomy (PCNL) for stones, and the rest consisted of previous non-stone interventions including pyeloplasty and ureteric reimplantation. There were also 8 cases (1.7%) of structurally abnormal kidneys including horseshoe kidney, dilated collecting system and solitary kidney.

**Table 1:** Demographics and clinical outcomes.

	Values are median (IQR [range]) or number (%)
No. treated cases	459
No. Male (%)	300/459 (65.4)
Patient age (yrs)	54.0 (44–66 [20–94])
No. Left side (%)	254/459 (55.3)
SSD; mm*	106.2 (84.8–122.8 [36–184])
No. stone location (%)	
Upper pole	41/459 (8.9)
Midpole	76/459 (16.6)
Lower pole	161/459 (35.1)
Renal pelvis and PUJ	60/459 (13.1)
Proximal ureter	39/459 (8.5)
Mid ureter	28/459 (6.1)
Distal ureter	17/459 (3.7)
VUJ	37/459 (8.1)
No. with ureteric stent present (%)	32/459 (7.0)
Measures of stone burden:	
No. of stones	
1	397/459 (86.5)
2	40/459 (8.7)
3	14/459 (3.1)
4–6	8/459 (1.7)
Major axis length; mm*	7.2 (5.6–9.6 [2.6–22.1])
Minor axis length; mm*	5.1 (4.0–6.6 [1.6–15.3])
Vertical axis length; mm*	7.1 (5.3–9.8 [0.2–29])
Volume(s); mm^3^†	137 (62–318 [6–2409])
Total no. of pixels*	69 (46–117 [12–533])
CTTA variables:	
Mean HU*	570 (430–700 [163–1217])
Standard deviation of the HU*	373 (267–453 [42–1069])
MPP*	572 (440–703 [163–1241])
Entropy*	4.2 (3.8–4.7 [2.5–5.8])
Kurtosis*	−1.1 (−1.3–−0.8 [−1.6–1.86])
Skewness*	0.32 (0–0.53 [−1.14–1.3])
No. SWL sessions	
1	202/458 (44.1)
2	134/458 (29.3)
3	54/458 (11.8)
≥4	69/458 (15)
No. follow-up imaging modality:	
X-ray	289/459 (63)
US	43/459 (9.4)
CT KUB	127/459 (27.7)
No. days of follow-up after final SWL treatment	124 (27–420 [0–1989])
No. completely stone free (%)	213/459 (46.4)
No. stone free with CIRFs	282/459 (61.4)

* Values were measured from the largest cross-sectional slice of the largest stone where more than one stone was treated. † Stone volume is the sum of individual stone volumes in cases with more than one stone. CIRFs, clinically insignificant residual fragments; CT KUB, computed tomography kidneys ureter bladder; CTTA, computed tomography texture analysis; HU, Hounsfield unit; PUJ, pelviureteric junction; MPP, mean of the positive pixels; SSD, skin-to-stone distance; SWL, extracorporeal shockwave lithotripsy; VUJ, vesicoureteric junction; US, ultrasound.

### SWL treatment and follow-up

Based on the information provided in the SWL treatment report of the first session, 85.7% of cases received 4000 shocks, and 51.4% received the full anticipated energy level of 6 or more during SWL. There was no statistically significant difference in success rates between cases which received 4000 shocks or energy level of 6 or more, and cases which received less than 4000 shocks or an energy level of less than 6. 90.9% of cases reported satisfactory or good pain tolerance, with 9.1% reporting poor pain tolerance during SWL. Based on the radiographer’s assessment of extent of fragmentation seen at time of SWL treatment, 27% reported no fragmentation seen, 30% reported possible fragmentation and 43% of cases showed clear fragmentation. There was no statistically significant difference in stone free rates between different degrees of pain tolerance reported, or different extents of fragmentation seen at the time of SWL.

The median length of follow up from the last SWL session was 124 days (IQR = 27–420). Of the 46.4% of cases with an outcome of ‘Completely Stone Free’, 56.6% required just one SWL session, 28.8% required two sessions, 7.1% required three sessions and 7.5% required four or more sessions.

### Univariate analyses

**Figure 1:**
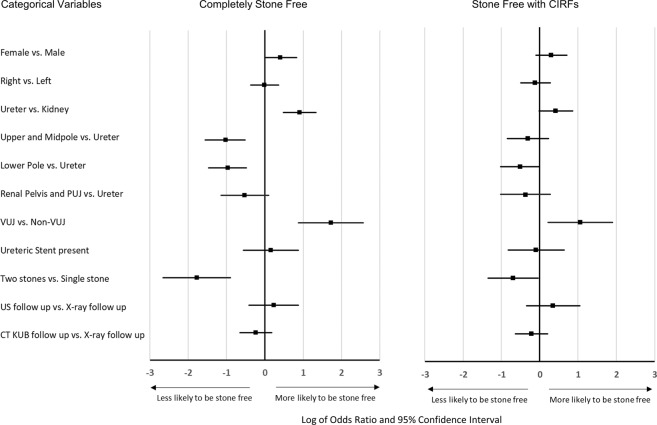
Forest plot of the log of the odds ratio and its 95% confidence interval for comparison of categorical predictor variables for the odds of having been ‘Completely Stone Free’ and ‘Stone Free with CIRFs’. The odds ratio refers to the first mentioned variable e.g. Female, and the second listed variable is the reference category e.g. Male. Therefore, Females have a slightly better odds of SWL success than Males. *CIRFs, clinically insignificant residual fragments; CT KUB, computed tomography of the kidneys, ureter and bladder; PUJ, pelviureteric junction; US, ultrasound scan; VUJ, vesicoureteric junction.*

**Figure 2:**
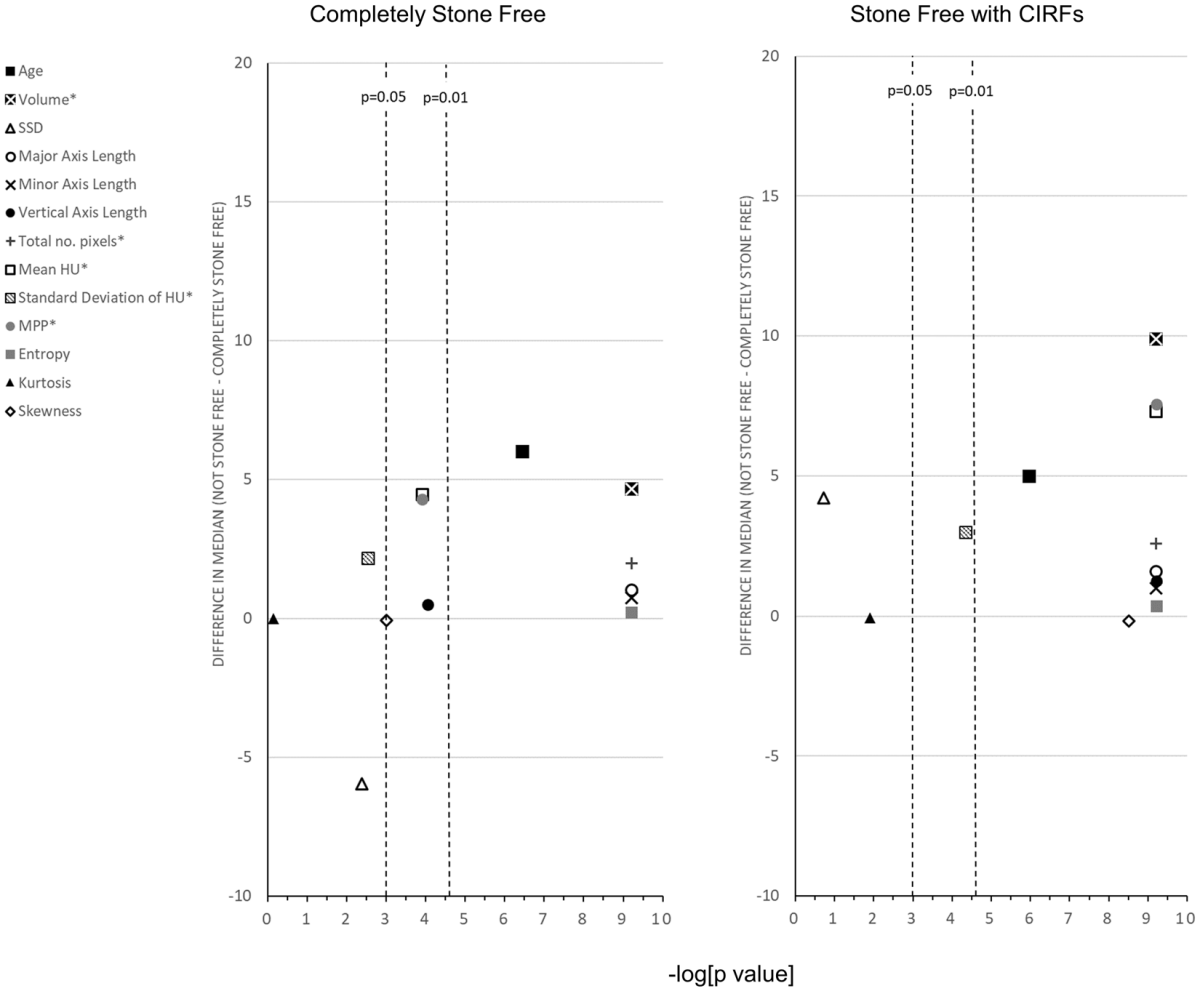
Volcano plot of the difference in median and the p-value of the Mann–Whitney U test for the comparison of continuous predictor variables between an SWL outcome of ‘Completely Stone Free’ and ‘Stone Free with CIRFs’. Variables labelled with * have had their median value divided by a factor of ten to allow representation of the ‘Difference in Median’ of all variables on the same scale axis. *CIRFs, clinically insignificant residual fragments; HU, Hounsfield units; MPP, mean of the positive pixels; SSD, skin to stone distance.*

Table 2, and Fig. 1 and 2, summarize the univariate analyses for both the outcomes of ‘Completely Stone Free’ and ‘Stone Free with clinically insignificant residual fragments (CIRFs)’. Variables associated with a significantly (p < 0.05) lower SWL success rate based on an outcome of ‘Completely Stone Free’ were: male gender, increasing age, larger stone size (based on all three axis measurements or stone volume), two or more stones in the same location, a stone located in the kidney compared to the ureter, a stone not in the vesicoureteric junction location, higher ‘mean HU’, ‘mean of the positive pixels’ ‘entropy’ and ‘total number of pixels’. Increasing SSD, presence of a stent, a lower pole location (compared to upper or midpole locations), and stone laterality did not result in a significantly lower SWL success rate.

**Table 2:** Univariate analysis of the pre-treatment factors, number of SWL sessions and follow-up imaging modality.

	Completely Stone Free Yes/No				Stone Free with CIRFs Yes/No			
	Yes	No	OR (95% CI)	p value	Yes	No	OR (95% CI)	p value
No. treated cases	213/459 (46.4)	246/459 (53.6)	—	—	282/459 (61.4)	177/459 (38.6)	—	—
Gender:								
Male	129/300 (43)	171/300 (57)	1	—	177/300 (59)	123/300 (41)	1	—
Female	84/159 (52.8)	75/159 (47.2)	1.49 (1.01, 2.29)	0.049	105/159 (66)	54/159 (34)	1.35 (0.91, 2.02)	0.159
Patient age (yrs)	51.0 (40–64)	57 (47–67)	—	0.0016	52.0 (41–65)	57.0 (48–67)	—	0.0026
Side of stone:								
Left	118/254 (46.5)	136/254 (53.5)	1	—	159/254 (62.6)	95/254 (27.4)	1	—
Right	95/205 (46.3)	110/205 (53.7)	1.00 (0.69, 1.44)	1.000	123/205 (60)	82/205 (40)	0.89 (0.61, 1.31)	0.630
SSD; mm*	109 (88–124)	102 (83–121)	—	0.0931	105 (86–120)	108 (84–125)	—	0.4843
Renal vs. Ureteric stones:								
Kidney (all sites)	137/338 (40.5)	201/338 (59.5)	1	—	199/338 (58.9)	139/338 (41.1)	1	—
Ureter	76/121 (62.8)	45/121 (37.2)	2.48 (1.62, 3.80)	<0.001	83/121 (68.6)	45/121 (31.4)	1.53 (0.98, 2.37)	0.065
No. stone location (%) compared to Ureteric Stones as the reference category:								
Upper and Midpole	44/117 (37.6)	73/117 (62.4)	0.36 (0.21, 0.60)	<0.001	72/117 (61.5)	45/117 (38.5)	0.73 (0.43, 1.25)	0.278
Lower pole	63/161 (39.1)	98/161 (60.9)	0.38 (0.23, 0.62)	<0.001	91/161 (56.5)	70/161 (43.5)	0.60 (0.36, 0.98)	0.048
Renal pelvis/PUJ	30/60 (50)	30/60 (50)	0.59 (0.32, 1.11)	0.111	36/60 (60)	24/60 (40)	0.69 (0.36, 1.31)	0.318
Ureteric stent present:								
No	197/427 (46.1)	230/427 (53.9)	1	—	263/427 (61.6)	164/427 (38.4)	1	—
Yes	16/32 (50)	16/32 (50)	1.17 (0.57, 2.40)	0.716	19/32 (59)	13/32 (41)	0.911 (0.44, 1.89)	0.852
Measures of stone burden:								
1 Stone	204/397 (51.4)	193/397 (48.6)	1	—	255/397 (64)	142/397 (36)	1	—
2 Stones	6/40 (15)	34/40 (85)	0.17 (0.07, 0.41)	<0.001	19/40 (48)	21/40 (52)	0.50 (0.26, 0.97)	0.041
Major axis length; mm*	6.7 (5.3–8.7)	7.7 (6.1–10.8)	—	<0.0001	6.7 (5.3–8.7)	8.3 (6.3–11.4)	—	<0.0001
Minor axis length; mm*	4.7 (3.8–6.1)	5.4 (4.2–7.2)	—	0.0001	4.7 (3.7–6.1)	5.8 (4.4–7.7)	—	<0.0001
Vertical axis length; mm*	6.9 (5.0–9.2)	7.4 (5.5–10.3)	—	0.0176	6.7 (4.8–9.0)	8.0 (6.0–11.1)	—	<0.0001
Volume(s); mm^3^†	109 (53–231)	156 (76–403)	—	<0.0001	102 (51–241)	201 (95–522)	—	<0.0001
Total no. pixels*	62 (37–101)	82 (49–140)	—	<0.0001	62 (37–101)	88 (53–153)	—	<0.0001
Measures of stone density and heterogeneity (CTTA variables):								
Mean HU*	543 (252–440)	587 (455–726)	—	0.0201	532 (413–664)	605 (481–759)	—	0.0001
SD of the HU*	364 (253–440)	385 (300–458)	—	0.0799	362 (250–440)	391 (316–464)	—	0.0129
MPP*	545 (416–680)	589 (461–732)	—	0.0202	535 (415–668)	610 (489–761)	—	0.0001
Entropy*	4.1 (3.6–4.6)	4.3 (3.8–4.8)	—	<0.0001	4.1 (3.6–4.6)	4.5 (3.9–4.9)	—	<0.0001
Kurtosis*	−1.11 (−1.29– −0.89)	−1.11 (−1.30– −0.82)	—	0.8733	−1.09 (−1.25– −0.87)	−1.14 (−1.33– −0.82)	—	0.1497
Skewness*	0.35 (0.08–0.52)	0.28 (−0.11–0.54)	—	0.0501	0.37 (−0.10–0.54)	0.20 (−0.16–0.51)	—	0.0002
No. SWL sessions^‡^	1.0 (1–2)	2.0 (1–4)	—	<0.0001	1.0 (1–2)	2.0 (2–4)	—	<0.0001
No. follow-up imaging modality:								
X-ray	136/286 (47.6)	150/286 (52.4)	1	—	177/286 (61.9)	109/286 (28.1)	1	—
US	23/43 (53)	20/43 (47)	1.27 (0.66, 2.41)	0.515	30/43 (70)	13/43 (30)	1.42 (0.71, 2.84)	0.398
CT KUB	53/127 (42)	74/127 (58)	0.79 (0.52–1.20)	0.286	72/127 (56.7)	55/127 (43.3)	0.81 (0.53–1.23)	0.329

Values are median (IQR) or number (%). Continuous variables underwent the Mann–Whitney U test and therefore only the p-value is presented. Categorical variables underwent the chi-square test with odds ratios expressed relative to the reference group (usually chosen as group with the largest number of cases), with asymptotic confidence intervals and Fisher’s exact p values. Odds ratios and p-values have not been included for groups with less than 3 observations, when split by outcome. * Values were measured from the largest cross-sectional slice of the largest stone in cases where more than one stone was treated. † Stone volume is the sum of individual stone volumes in cases with more than one stone. CIRFs, clinically insignificant residual fragments; CT KUB, computed tomography kidneys ureter bladder; CTTA, computed tomography texture analysis; HU, Hounsfield unit; PUJ, pelviureteric junction; SD standard deviation; SSD, skin-to-stone distance; SWL, extracorporeal shockwave lithotripsy; US, ultrasound scan, VUJ, vesicoureteric junction.

The significant (p < 0.05) predictor variables on univariate analysis for the outcome of ‘Stone Free with CIRFs’ are the same as for the outcome of ‘Completely Stone Free’ except for the texture analysis variables of Standard Deviation of the HU (which was significantly higher for the unsuccessful cases) and Skewness (which was significantly lower in the unsuccessful cases).

### Multivariable analyses

LASSO (least absolute shrinkage and selection operator) analysis was first performed without the inclusion of CTTA variables. Table 3 shows the variables chosen in this initial model which were: sex, age, number of stones in the same location, length of the major axis, length of the vertical axis, SSD and stone location in the vesicoureteric junction (VUJ). Of note here, SSD and vertical axis size contributed an effect in the opposite direction to that expected: higher variable values resulted in a greater likelihood of a stone free outcome. However, along with age, these three variables had a very small effect on the probability of the outcome with coefficients of <0.1. This model had an AUC (area under the curve) of 0.66 on ROC (receiver operator curve) analysis and a Hosmer-Lemeshow p value of <0.001 indicating the model is poorly calibrated. Confirmation using Random Forests approach yielded a similar AUC of 0.67.

**Table 3:** Results of multivariable analysis using the LASSO method for the outcome of ‘Completely Stone Free’ showing chosen predictor variables and their corresponding coefficients.

Variable	LASSO co-efficient
(Intercept)	4.5
Sex = Male	−0.582
Age	−0.006
Number of stones being treated	−0.817
Size of the major axis (of the largest stone)	−0.176
Size of vertical axis (of the largest stone)	0.002
Skin-to-stone distance	0.004
Stone location not in the VUJ	−0.751
**ROC analysis of model:**	AUC = 0.66
**At a predicted probability cut-off of 0.51 (for a ‘Completely Stone Free’ successful outcome of SWL treatment):**	
Sensitivity = 59.8%	
Specificity = 56.3%	

Variables used as input into this model were those who had a significance of p > 0.1 on univariate analysis. The chosen predictor variables shown in this table with a negative coefficient indicate that higher values are linked to greater probability of unsuccessful SWL with outcome of ‘Not Completely Stone Free’. The LASSO co-efficient is not standardized and relates to the values of the variables and not the relative weight of influence of the variables. For example, for every one-year increase in age, there is a 0.006 percentage points decrease in the predicted probability of SWL success. The dataset was split into 2/3 training data to develop the predictive model whose performance is evaluated on the remaining 1/3 test data for ROC analysis. SWL, extracorporeal shockwave lithotripsy; LASSO, least absolute shrinkage and selection operator; ROC, receiver operator curve; VUJ, vesicoureteric junction.

Table 4 summarizes the results of all the multivariable analyses and shows that, the ability of the LASSO or Random Forests models to correctly classify a case for both ‘Completely Stone Free’ and ‘Stone Free with CIRFs’ outcomes of SWL, was moderate, with AUCs of 0.64 to 0.67 produced from ROC analysis. Although this model was not reliable in predicting for successful SWL cases based on this ROC analysis, it could help identify those cases most likely to fail SWL treatment with a negative predictive value of 84.3% (when using a predicted probability cut-off of 0.29 and sensitivity and specificity threshold of 95.8% and 19.5% respectively).

**Table 4:** Comparison of the performance of predictive models for the outcome of ‘Completely Stone Free’ or ‘Stone Free with CIRFs’ using three methods of multivariable analyses; first without the inclusion of CTTA variables and then with the addition of CTTA variables to the models.

		Outcome			
		Completely Stone Free		Stone Free with CIRFs	
		Patient and stone related variables	With the addition of CTTA variables	Patient and stone related variables	With the addition of CTTA variables
**Multivariable models evaluated**	**Statistic of model performance**				
LASSO	Area under the curve on ROC analysis for discrimination performance	0.66	0.64	0.67	0.67
	Hosmer-Lemeshow p-value	p < 0.001	p < 0.001	p < 0.001	p < 0.001
Partial Least Squares	Q2 (quality assessment) statistic	0.117	0.086	—	—
Random Forests	Area under the curve on ROC analysis	0.67	0.65	—	—

A significant Hosmer–Lemeshow p-value, as shown in this table, indicates the model is poorly calibrated. Partial Least Squares and Random Forests methods were not performed for the outcome of ‘Stone Free with CIRFs’. CIRFs, clinically insignificant residual fragments; CTTA, computed tomography texture analysis; LASSO, least absolute shrinkage and selection operator; ROC, receiver operator curve.

There was no additional benefit to the predictive ability of the LASSO model after CTTA variables were included (Table 4). This was confirmed using partial least squares (PLS) analysis, which showed a reduced quality assessment (Q2) statistic from 0.117 to 0.086, once CTTA variables were added to the model. LASSO analysis of a subset of the data of solitary stones only (n = 371) produced an AUC of 0.63 for the outcome of ‘Completely Stone Free’ and an AUC of 0.75 for the outcome of ‘Stone Free with CIRFs’, with no improvement on predictive ability after the addition of CTTA variables.

## Discussion

This study aimed to evaluate the performance of a model to predict for the outcome of SWL treatment using both patient and stone related variables. The resulting model showed moderate predictive ability (AUC of 0.64–0.67) in terms of the ability to discriminate between a successful or unsuccessful outcome after SWL treatment. Predictive ability in terms of calibration was poor based on a significant Hosmer–Lemeshow test, meaning that the observed and expected outcomes in tested subgroups of our sample were not similar. Including variables calculated from CTTA did not increase the predictive ability of the model. There could be several reasons for this including bias in the data collection and classification of outcome, and not having selected the most influential factors for SWL efficacy as variables in the model. Our results suggest there is not enough current understanding of the important predictive factors for SWL efficacy to be able to produce a useful model to aid clinical decision making for which cases are most suitable for SWL treatment.

This is the first study that has examined a wide range of both patient and stone related variables using three different methods of multivariable analyses. Several studies have found the significance of single factors to predict for SWL success^4,11,15,20,23^. Of the studies which produced a predictive model using a combination of different factors thought to affect SWL success rate^12,17–19,28^, only three studies (which both used logistic regression) have presented ROC analysis on the predictive performance of these models^10,16,21^. Our AUC values (0.64–0.67) were lower than the AUC of 0.75 to 0.87 found using logistic regression models in these three previous studies^10,16,21^ - even though there were variables in agreement between our study and the above three studies, in terms of finding that age, SSD, stone size and number of stones were significant predictors in a multivariable model. This may be explained due to the problems of overfitting and the inclusion of related or co-correlated variables when using logistic regression. The LASSO method reduces this problem by shrinking the weight of each predictor, and the PLS method also has the advantage of not using univariate results for the pre-selection of variables into the multivariable analysis. These methods used in our study therefore reduces the overestimation of variable significance, and overall predictive ability. We also kept continuous data as continuous (to avoid loss of information through categorization which three other studies have done with some variables before statistical analysis)^10,13,16,28^.

Furthermore, unlike previous studies, we also performed several multivariable analyses. A comparison of the performance of predictive models for the outcome of ‘Completely Stone Free’ or ‘Stone Free with CIRFs’ using three methods of multivariable analyses in Table 4, shows that, neither the LASSO method nor the Random Forests method produced an AUC of more than 0.7. At this predictive level, the models produced in this study are unlikely to be useful in discriminating for cases most likely to succeed or fail SWL.

### Use of CTTA on stone imaging

Recent interest in extracting more information from CT images of the stone have produced results suggesting the importance of stone heterogeneity, in addition to stone attenuation, as a predictive factor for successful SWL^14,15,22–25,28^. Stone attenuation is a well-researched predictive factor for SWL outcome. Stone attenuation, as measured in this study using the mean HU value, was a significant predictor for SWL outcome on univariate analyses but was not chosen to be included in the multivariable analyses, suggesting that size may have had more weight in our model. Previous studies have used a variety of methods to quantify stone heterogeneity from use of subjective visualization to statistical methods^14,15,22–25,28^. The CTTA method used in this study calculates variables which have been previously validated in tumor imaging^26^. This method also reduces measurement bias as there is no user-dependent variability in drawing the region of interest (ROI) or interpreting the results. Our study has the advantage of using objective measures of stone heterogeneity by using textural analysis of the distribution of all of the pixels in a cross-section of stone, rather than subjective observation of stone appearance on CT which can be difficult to differentiate^14,23^. One study found that they were unable to view the internal structure of stones on non-contrast CT to classify as hyper-or hypodense centre or homogeneous^19^. Rather than subjecting patients to higher resolution CT scans, textural analysis may provide a more practical way of assessing stone heterogeneity. However, in our study, the addition of textural analysis variables did not significantly improve the predictive ability of the multivariable model, although many textural features were significant on univariate analysis. It is likely that variables related to the size of the stone, including total number of pixels, and entropy which is correlated to stone size, are the most influential factors. However, as methods of CT image analysis develop, it is foreseeable that we will gain more information on stone characteristics, including architecture and composition to aid treatment.

### Study limitations

Limitations of our study include: its retrospective nature and the measurement of some variables on the largest stone only if there was more than one stone. Retrospective collection of variable and outcome data may have led to bias and reduced the predictive ability of a multivariable model. However, this also allowed a more pragmatic approach, by including cases with more than one stone and repeated SWL sessions, our results may be more applicable to clinical practice. A previous study has found that the size and HU density of the largest stone was a better predictor than the mean where more than one stone was treated^18^. Our analysis of the solitary stones showed similar predictive ability and suggests that our measurements based on the largest stone have not biased the data.

In summary, analysis of clinical and stone imaging factors, including more novel variables of CTTA in this study has not produced a useful model for predicting the outcome of SWL. This study supports findings from previous studies on the importance of predictor factors relating to patient age and stone size, as well as contradicting some popular beliefs on the importance of skin-to-stone distance and the lower pole position. However, our results do not support previous study findings which suggest CTTA variables have additional predictive value above traditional factors related to stone size.

## Methods

Data was analyzed from a single center in the UK in accordance with the relevant guidelines and regulations as set out by the approving body, the Health Research Authority, United Kingdom (ethics committee reference 16/HRA/6001). We used an existing anonymized database that was prospectively entered for all patients undergoing SWL, at a single site, available between 2011 and 2016. Individual patient consent was not required as no patient identifiable records were obtained in this study. Inclusion criteria were availability of a pre-treatment CT, and available follow up imaging. This identified 459 consecutive cases (from 420 patients). Each case consisted of a course of SWL treatment focused on one or more stones in a single location in the kidney or ureter. CT scans were performed on a multidetector row helical CT scanner (LightSpeed plus, General Electric Medical Systems, USA) and reconstructed at 1.25 mm slice thickness. SWL was performed using a Storz Modulith SLX F2 lithotripter (Storz Medical AG, Switzerland) by an experienced radiographer. The aim was to deliver 4000 shocks at 2 Hz for each treatment session at energy levels recommended by the manufacturer, and according to pain tolerance. At two weeks after the first two SWL treatments, stone clearance was reviewed using a plain x-ray (or US or CT kidneys ureters bladder if not visible on follow-up x-ray) by a urologist and radiologist to decide further management. If stone fragments were still visible, patients were offered either more SWL or other treatment options. All follow up information relevant to the stone being treated was collected from the date of the last SWL treatment to 2017.

### Patient and imaging variables

**Figure 3:**
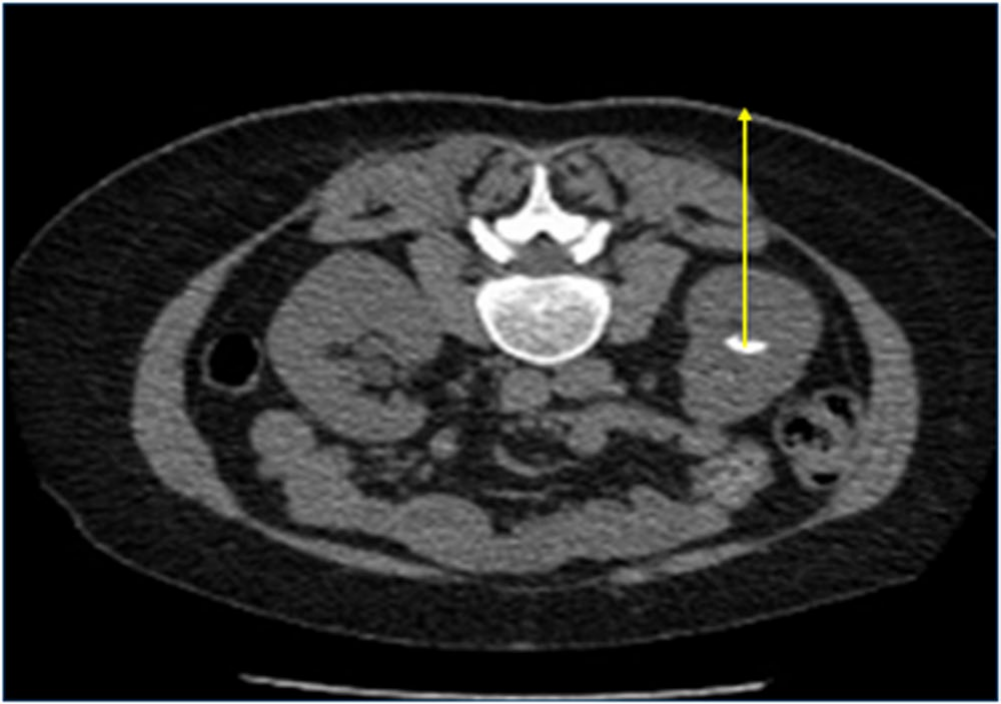
Measurement method of skin-to-stone distance from the centre of the stone to the posterior skin, in line with the angle of the spinous process (yellow arrow).

Supplementary Information Table-A includes a list of the patient and imaging related variables included in the statistical analysis, with descriptions of the methods of measurement. The patient variables included were: patient age, body mass index, skin-to-stone distance (SSD), and, for the initial SWL treatment session, the pain tolerance, energy level reached in joules, number of shocks given and radiographer comment of whether fragmentation was seen during SWL. Measurements of stone size and SSD were taken using radiographic calipers on a workstation (Advantage Windows 4.0, GE Medical Systems). SSD was taken as the distance between the centre of the stone and the skin at 90° (or parallel to the line of the vertebral spinous process) using radiographic calipers (Fig. 3). This method was chosen to reflect the path of the SWL beam during treatment.

The stone variables included were: major, minor and vertical axis length (measured on the image slice containing the largest cross-sectional diameter of the axis of interest, noting that this may not represent the ‘real’ axes of the stone if this does not coincide with the axial view), number of stones being treated in the same location, volume of stone(s) and stone location (categorized as upper pole, midpole, lower pole, renal pelvis, pelviureteric junction (PUJ), proximal ureter, midureter, distal ureter or VUJ). The volume of the stone was measured using the ellipsoid formula^29^, and if there was more than one stone being treated in the same SWL session, the volumes of all stones were summed.

**Figure 4:**
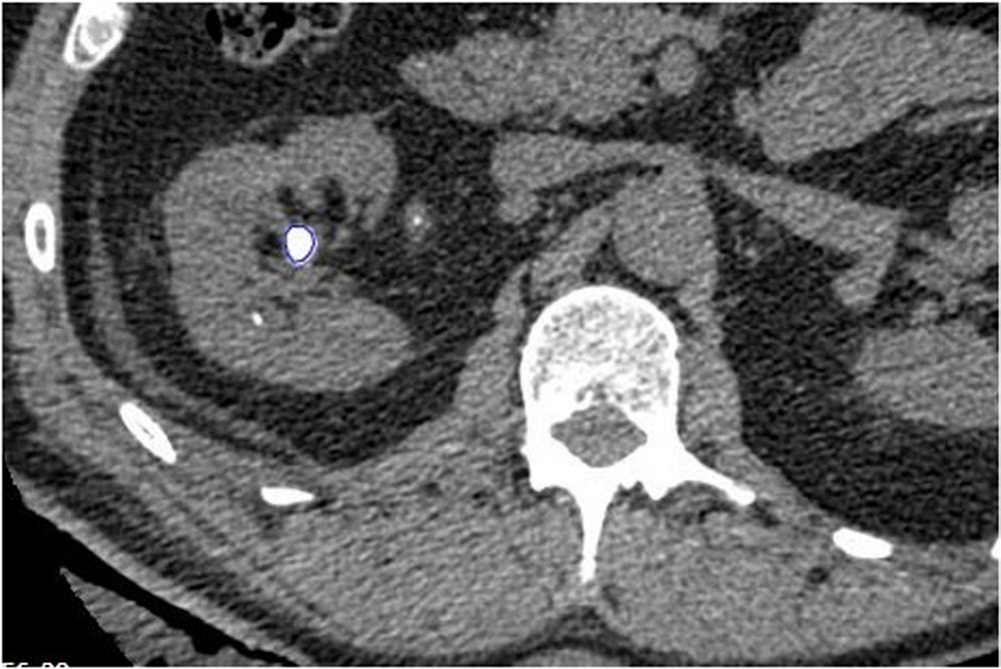
Example of a Region of Interest (ROI) automatically drawn (in blue) using CT textural analysis software (CTTA) to fit the largest cross-sectional area of the largest stone being treated by shockwave lithotripsy.

To improve characterization of stone heterogeneity as a predictor variable, we have used a CT textural analysis (CTTA) software program (Stone Checker Software Limited, Radstock, UK). This software includes a filtration-histogram technique validated for measuring tumor heterogeneity^27^, which we have also previously tested on kidney stones^22^. For calculation of CTTA variables, a region of interest was automatically fitted to just inside the outline of the stone using the CT image slice with the largest cross-sectional diameter (Fig. 4). The following stone variables were measured using the software: mean HU, standard deviation of the HU, mean of the positive pixels (MPP) present, entropy, skewness, kurtosis, and the total number of pixels present. The variables of MPP, skewness and kurtosis were calculated through a process of analyzing histograms of the HU values of all the pixels.

### Outcome of treatment

Two definitions of successful outcome of SWL were used in this study: 1) ‘Completely Stone Free’ 2) ‘Stone free with clinically insignificant residual fragments (CIRFs)’ ≤ 4 mm. Outcome was determined by radiologist review of the follow-up imaging modality of either x-ray, US or CT KUB, and confirmed with patient records that no further treatment was required. For cases with only US or x-ray follow up imaging (which are known to overestimate stone free rate compared to CT follow-up), *and* had required more than 3 sessions of SWL treatment, were classified as failures of treatment. This aimed to reduce the measurement bias of having different imaging modalities in determining the outcome measure. The length of follow-up was measured from the last SWL session attendance.

### Statistical analysis

Summary statistics of the continuous factor variables were presented as medians as the distributions could not be assumed to be normal, and therefore nonparametric Mann Whitney U test was performed for univariate analysis to test for difference between the groups of successful or unsuccessful outcome of SWL treatment. Categorical variables underwent the Chi-square test. Multivariable analysis using the pre-treatment variables was performed using the LASSO (Least absolute shrinkage and selection operator) regression analysis method^30^. This approach combines variable selection with shrinkage of the weights of each variable, which gives better prediction when using a large number of predictor variables. All predictor variables which were significant at p < 0.1 were considered in the LASSO analysis. This method does not produce p values but does produce the weighted coefficients of each variable in the model. The discrimination, and calibration, ability of the LASSO model was evaluated using ROC analysis and the Hosmer–Lemeshow goodness of fit test respectively.

In addition to the LASSO method, the Partial Least Squares and Random Forests approach was used to further ensure our conclusions were robust. Partial Least Squares finds an optimal weighted combination of predictor variables to discriminate between two groups. This weighted combination is based on all supplied variables, meaning there is no pre-selection of variables to enter into the model, unlike LASSO. It is designed for situations with a large number of variables and fewer observations. Random Forests method selects repeated bootstrap samples from the data set and creates a decision tree at each node where an optimum variable to split is selected. Each node uses only a subset of the variables. Predictions are obtained by consensus over bootstraps and applied to a holdout test sample. Our sample size was deemed adequate in this study by the statisticians to support the number of variables being investigated for multivariable analysis. All statistical analysis was performed by Exploristics Ltd. (Belfast, UK) using R (R Foundation for Statistical Computing, Vienna, Austria)^31^.

### Ethical approval

Ethical approval was obtained from the Health Research Authority in the United Kingdom for this study protocol. Local hospital research and development approval was also obtained.

## Supplementary information

Supplementary Information Table-A

## Data Availability

The datasets generated during and/or analyzed during the current study are available from the corresponding author on reasonable request.
