# Supplementary material for: Predicting shockwave lithotripsy outcome for urolithiasis using clinical and stone computed tomography texture analysis variables
Source: Sci Rep. 2019 Oct 11;9:14674. doi: 10.1038/s41598-019-51026-x (PMC6788981; doi:10.1038/s41598-019-51026-x)
Supplement: Supplementary file 1 — Supplementary Information Table-A [file 41598_2019_51026_MOESM1_ESM.pdf]

Supplementary Information

Title Page

Predicting shockwave lithotripsy outcome for urolithiasis using clinical and stone computed tomography texture analysis variables.

Cui HW, Silva MD, Mills AW, North BV, and Turney BW.

Supplementary Information

Table-A Description of the variables included in this study for statistical analysis.

Variable Name	Description of variable	Method of measurement / Expected range
**Patient related variables**		
Age	Age at time of first SWL treatment	Years
Sex	Gender of patient being treated	
Presence of ureteric stent	Presence of absence of ureteric stent in the kidney being treated with SWL.	Ureteric stent seen on patient imaging during course of SWL treatment.
Skin-to-stone distance (SSD)	Distance in mm between the skin to the centre of the largest stone being treated by SWL.	SSD was taken as the distance between the centre of the stone and the skin at 90° (or parallel to the line of the vertebral spinous process) using radiographic calipers (see Fig. 3). This method was chosen to reflect the path of the SWL beam during treatment.
Body mass index (BMI)	Measured as kg/m^2	
Pain Tolerance	Perceived pain tolerance of the patient by the treating radiographer at the time of the first SWL treatment.	Reported as one of three levels: poor pain tolerance, satisfactory pain tolerance and good pain tolerance.
Highest energy level reached	Energy levels available for delivery by the lithotripter ranged from 1 to 9. Usually level 4 was used for renal stones, and level 6 or 7 for ureteral stones.	Energy level as reported by the Storz Modulith lithotripter.
Number of shocks delivered	Total number of shocks delivered at first session of SWL, with an aim to deliver 4000 shocks.	Number of shocks delivered as reported by the Storz Modulith lithotripter.
Extent of fragmentation seen	Extent of visible fragmentation as reported by the treating radiographer based on fluoroscopic images at the time of the first SWL treatment.	Reported as one of three level: no fragmentation seen, some fragmentation seen, or clear fragmentation seen.
**Stone related variables**		
Major axis length	Length in mm of the major axis, defined as the cross-sectional diameter of the stone on axial view.	Measured using radiographic calipers on the axial CT image of the largest cross-sectional slice of the largest stone.
Minor axis length	Length in mm of the minor axis, defined as the smallest diameter of the stone in the axis that is perpendicular to the major axis on axial view.	
Vertical axis length	Length in mm of the vertical axis, defined as the largest diameter of the stone on coronal view.	
Maximum axis length	Length in mm of the largest axis of measured out of the major, minor and vertical axes.	
Number of stones	The discrete number of stones being treated in the same location during a course of SWL.	Number of stones as reported by the radiologist in the pre-treatment CT scan.
Volume		The volume of each stone was measured using the ellipsoid formula by inputting the major, minor and vertical axes measured as described above (volume = π / 6 * (major axis * minor axis * vertical axis). If there was more than one stone in the same

		location being treated, the sum of the individual stone volumes was used to represent the total stone burden at the time of SWL.
Laterality	Location of the stones being treated by one course of SWL in the left or right kidney.	Based on the CT scan report.
Stone Location	Location of the stone as determined by the authors on the pre-treatment CT scan.	Location was classified as one of the following nine categories: upper pole, midpole, lower pole, renal pelvis, pelviureteric junction, proximal ureter, mid-ureter, distal ureter, and vesicoureteric junction.
**Stone related variables measured using CT texture analysis (CTTA) software**		
Mean HU	The mean Hounsfield unit value of all the pixels in the ROI.	CTTA variables were calculated by the software, based on distribution of all of the pixels included within a region of interest (ROI).<br><br>This ROI was automatically fitted by the software to have an outline just within the border of the stone (Fig. 4). The ROI was created using the CT axial image slice of the largest cross-sectional area of the largest stone being treated by SWL.
Standard deviation of the HU	Standard deviation of the HU within the ROI.	
Mean of the Positive Pixels (MPP)	Mean HU value of all of pixels with HU values of 0 or greater within the ROI.	
Entropy	Entropy, as a measure of the randomness, of the distribution of the HU values of the pixels within the ROI.	
Skewness	Skewness measured the degree of symmetry of the distribution of the HU of all pixels in the ROI. A negative skewness value means the distribution is skewed left and the left tail is long relative to the right tail. This would mean more of the data is located towards the higher HU values.	
Kurtosis	Kurtosis measured the combined weight of the tails of the distribution of the HU values of the pixels, relative to the rest of the distribution. A negative kurtosis means lighter tails, with more of the HU centrally distributed rather than at the extremes of HU value.	
Total number of pixels	This is the total number of pixels counted within the ROI and is representation of the cross-sectional area of the stone.	
